# Stage-based treatment for thymoma in due consideration of thymectomy: a single-center experience and comparison with the literature

**DOI:** 10.1186/s12957-015-0718-z

**Published:** 2015-10-16

**Authors:** Joerg Lindenmann, Nicole Fink-Neuboeck, Martin Pichler, Udo Anegg, Alfred Maier, Josef Smolle, Freyja Maria Smolle-Juettner

**Affiliations:** Division of Thoracic and Hyperbaric Surgery, Department of Surgery, Medical University Graz, Auenbruggerplatz 29, 8036 Graz, Austria; Division of Oncology, Department of Internal Medicine, Medical University Graz, Graz, Austria; Institute of Medical Informatics, Statistics and Documentation, Medical University Graz, Graz, Austria

**Keywords:** Thymoma, Surgery, Thymectomy, Myasthenia gravis, Survival

## Abstract

**Background:**

Thymomas represent an uncommon and heterogeneous group of intrathoracic malignancies which require different treatments corresponding to their individual tumor stage. The objective of this study was to review the efficacy of our applied stage-based treatment for thymoma in due consideration of thymectomy.

**Methods:**

This is a single-center, institutional review board-approved retrospective study of 50 consecutive patients with thymoma treated at our division within 10 years.

**Results:**

There were 29 women (58 %) and 21 men (42 %), mean age 58.3 years. Twenty nine (58 %) had clinical symptoms and 14 (28 %) had myasthenia gravis. Forty-five patients (90 %) underwent thymectomy and complete resection was done in 42 cases (93.3 %). Histologic results were 6 subtype A, 5 AB, 8 B1, 12 B2, 12 B3, and 7 C. The Masaoka staging system revealed 20 stage I, 18 stage II, 6 stage III, and 6 stage IV. Two patients had neoadjuvant therapy and 25 received postoperative treatment. Five (11.1 %) had tumor recurrence, treated with re-resection. The 5-year disease-free survival was 91.5 %. Two patients died of tumor progression and three died of other causes (10 %). The 5-year overall survival was 82.3 % and the median survival time was 92.1 months. The 5-year survival rate after thymectomy was 87.2 % and the median survival was 92.1 months.

**Conclusions:**

Complete resection still remains the mainstay in the treatment of non-metastatic thymoma and should be performed whenever feasible. Close multidisciplinary teamwork is mandatory to optimize the neurologic outcome and to prolong postoperative survival.

## Background

Thymomas, which originate from the epithelial cells of the thymus, are uncommon thoracic malignancies with an annual incidence of 0.15/100,000 persons. Thymomas are the most common tumors of the anterior mediastinum, representing 50 % of all cases in adults [1, 2]. Thymomas develop among all ages with a mean age of about 53 years [1]. There is nearly an equal gender distribution, although myasthenia gravis (MG) seems to occur more frequently in the younger collective and among female patients [3].

In one third of all cases, thymomas cause no clinical symptoms. Clinical symptoms develop due to tumor growth with subsequent compression, expulsion, or infiltration of the surrounding organs. MG, representing the most commonly associated parathymic syndrome, develops in one third of the cases [4]. In general, thymomas usually grow slowly. With increasing malignant potential, they grow rapidly and have a tendency to infiltrate surrounding tissues as well as locally spread mainly to the pleura, the pericardium, and the lungs. Distant metastases are uncommon and almost confined to the liver and the bones. Moreover, thymomas have a tendency for late local recurrence even after complete thymectomy [3, 5].

Even though thymic carcinomas are designated as type C tumors according to the World Health Organization (WHO) criteria, they are still different from thymomas [1]. They present a distinct group associated with high malignancy and therefore more aggressive behavior. Thymic carcinomas frequently attempt to infiltrate the surrounding tissue and metastasize to regional lymph nodes as well as distant sites [1, 3]. Due to the fact that thymic carcinomas represent a heterogeneous group with very low incidence (<10 % of thymic neoplasms) [3], they are not specially outlined in this study which focuses generally on the treatment and outcome of thymomas in general according to WHO criteria.

In particular in local advanced disease of thymoma, the multidisciplinary approach consisting of thymectomy, chemotherapy, and radiotherapy gains increasing importance [1–3].

Therefore, the objective of our study was to review the efficacy of our applied stage-based treatment for thymoma in due consideration of thymectomy.

## Methods

### Patients

Between 2003 and 2013, 51 consecutive patients suffering from histologically proven thymoma were treated at our division. One patient with thymoma subtype AB, who was not fit for surgery, died from pneumonia 3 months after diagnosis and was therefore excluded from the study. Finally, the complete records of 50 patients were available for evaluation and included into this study.

In particular, patient’s age, gender, functional assessment, the incidence of pre- and postoperative MG, clinical symptoms, histology, tumor staging, surgical aspects, complications, postoperative therapy, tumor recurrence, and disease-free survival (DFS) as well as overall survival (OS) were assessed. The data were collected prospectively in the database of the hospital and evaluated retrospectively. Survival data were obtained from the medical records and the database, by telephone interview with the patients and their family doctor, or by contacting the responsible registry office. However, OS was defined as the time from the date of surgery to the date of death or the date of last contact alive, respectively. DFS was calculated from the date of surgery to the date of diagnosis of tumor recurrence. This study was approved by the local ethics committee of the Medical University of Graz (number: 25-548 ex 12/13). As this is a retrospective non-intervention study, the institutional review board waived the need for written informed consent from the patients.

Functional evaluation of the patients included echocardiogram, cardiac ultrasound, and spiroergometry. The oncological staging consisted of chest X-ray; computed tomography (CT scan) of the thorax, the mediastinum, and the abdomen; and positron emission tomography-CT (PET-CT). Moreover, every patient, irrespective of underlying MG, was provided with neurological investigation before initiation of treatment.

Preoperative tumor biopsy enabling adequate staging was performed in the majority of patients. The histological assessment was done according to the commonly used WHO criteria [6].

In those patients with clearly visible encapsulated tumor of small or moderate size, the biopsy was not required. Preoperative tumor biopsy was performed in cases with strong suspicion of high malignancy corresponding to thymic carcinoma, in case of tumorous infiltration, and to rule out mediastinal lymphoma.

### Specific treatment

Based on both, the histologic results of the tumor biopsy and the CT scan, the preoperative tumor stage was assessed according to the most widely used Masaoka staging system [7], which still represents the chief staging criteria for thymomas. The patients underwent further specific treatment tailored to their particular tumor stage according to the decision made within the local interdisciplinary tumor board.

In case of local disease with completely encapsulated thymoma corresponding to Masaoka stages I and II, the patients were immediately provided with complete surgical resection.

In case of locally advanced disease corresponding to Masaoka stage III with high suspicion of tumorous infiltration into the surrounding tissue, neoadjuvant chemotherapy and/or radiotherapy was scheduled.

However, in case of very advanced disease with distant metastases corresponding to Masaoka stage IVB, surgery was not indicated and palliative chemotherapy and/or radiotherapy was therefore scheduled.

### Thymectomy

Thymectomy was performed using three surgical techniques: the trans-sternal and the trans-thoracic thymectomy represented the open approaches. The minimally invasive technique, video assisted thorascopic surgery (VATS), was also employed.

The trans-sternal approach was primarily provided for large-sized tumors, tumors with histologically proven high-grade malignancy, thymic carcinoma, radiologically verified broad tumorous adherence to adjacent structures expecting extended resection, and strong suspicion of tumor infiltration (locally advanced disease) and preceded neoadjuvant treatment.

The trans-thoracic approach was done in the minority of all cases. It was provided for completely encapsulated thymomas of large size strictly located in one hemithorax. Moreover, this approach was also used after anterior parasternal mediastinotomy for subsequent ipsilateral thymectomy within the same surgical intervention.

The minimally invasive approach, single-sided or double-sided VATS, was provided for completely encapsulated thymomas of small or medium size without evidence of tumor infiltration and without neoadjuvant therapy. Besides those facts, intraoperative conversion to the open approach was required in case of complication, technical failure, large tumor size, deficient overview, dense adhesions, or due to unexpected locally advanced disease with perithymic invasion.

### Trans-sternal approach

After median sternotomy, the entire anterior mediastinum was visualized. The phrenic was clearly identified on each side, and meticulous dissection was started on the left side at the lower border of the tumor onto the pericardium at the diaphragmatic sulcus, respectively. The dissection was continued along the course of the left phrenic nerve towards the innominate vein. Care was taken not to damage the phrenic nerve. Blunt dissection was continued onto the anterior and lower circumference of the innominate vein. The originating thymic veins were clipped, and dissection was continued towards the superior vena cava.

After mobilization of the superior portion of the thymoma, further dissection was continued along the course of the contralateral phrenic nerve until the diaphragm was reached. The entire thymoma, along with the residual four lobes of the thymus, associated with the mediastinal and pericardial as well as diaphragmatic fat pad, was resected en bloc. Finally, the extent of the en bloc resection represented the area between the course of both phrenic nerves as well as from the diaphragm to the thyroid gland, respectively.

In the case of local advanced disease with tumor infiltration into neighboring organs and tissues, extended resection of those affected structures was required. Removal of tumor infiltration into the adjacent lung parenchyma was accomplished by wedge resection using mechanical staple devices.

In case of involvement of the innominate vein, the affected part of the vascular wall was tangentially resected and tight sutures were applied.

In case of partial pericardial and/or diaphragmatic resection, the defect was closed using synthetic prostheses (GORE-TEX®, Flagstaff, AZ, USA) which were fixed with interrupted single-knot sutures. Furthermore, the pericardial patch was fenestrated to avoid postoperative cardiac tamponade.

### VATS approach

After maintenance of required single lung ventilation, three trocars, located at the typical positions, were inserted through the appropriate hemithorax and the ascertainable part of the mediastinum was visualized. The minimal-invasive dissection was performed in the same manner as mentioned above. After complete dissection, the resected specimen was placed into a bag and removed through the port. In case of larger tumor size associated with thymic branches reaching the contralateral pleura requiring bilateral VATS, the minimal-invasive approach was stopped after sufficient tumor dissection at the first site and the ports were closed as mentioned above. The VATS procedure was immediately repeated in the same manner on the contralateral side completed by successful retrieval of the en bloc-resected specimen within the bag through the port.

### Trans-thoracic approach

After maintenance of required single lung ventilation, the chest was opened through a muscle-sparing antero-lateral mini-thoracotomy. After visualization of the mediastinum, en bloc resection was performed as mentioned in the trans-sternal approach.

### Postoperative follow-up

After thymectomy, the decision concerning the adequate postoperative treatment based on Masaoka staging [7] was made within our local multidisciplinary tumor board consisting of thoracic surgeons, oncologists, radiotherapists, and pathologists. In the majority of cases, the oncological follow-up consisting of thoraco-abdominal CT scan was scheduled every 6 months for the first two postoperative years, and then annually for the following 3 years.

## Results

Among those 50 patients, the slight majority were women (58 %). The mean age was 58.3 years (range 22–82 years). At the time of admission, 29 patients (58 %) complained about symptoms.

Preoperative tumor biopsy was performed in the majority of patients, carried out by CT scan-guided fine needle aspiration (*N* = 12) or by using anterior parasternal mediastinotomy (*N* = 14), thoracoscopy (*N* = 5), muscle-sparing mini-thoracotomy (*N* = 3), or mediastinoscopy (*N* = 1), respectively.

At the time of diagnosis, 39/50 patients (78 %) had no suspicion of locally advanced disease. Distant metastases at one or more sites were diagnosed in five patients (10 %) affecting the lung, the bones, and the pleura. Neoadjuvant therapy, due to considerable local tumor infiltration, was administered in two out of those five patients (chemotherapy and chemoradiation).

Thymectomy was performed in 90 % of all patients. Extended resection due to locally advanced disease was required in 5/45 patients (11.1 %). The affected structures were lung, pericardium, phrenic nerve, innominate vein, and parietal pleura. However, intraoperative conversion from the VATS procedure to the open approach was required in seven cases. Among those 45 patients who had undergone surgery, 25 (55.6 %) received adjuvant treatment.

Histologic examination according to the WHO criteria revealed thymoma B2 and B3 as the predominating histologic subtype. Tumor staging according to the Masaoka system yielded stage I as the most frequent stage. The mean shortest diameter of the resected specimen was 6.5 cm (ranged 2.0 to 15.0 cm), whereas the mean largest diameter revealed 8.7 cm (ranged 4.0 to 19.0 cm). The mean volume was 357.1 cm^3^ (median 221.3 cm^3^). These clinicopathological parameters mentioned above are summarized in detail in Table 1.

**Table 1:** Detailed clinicopathological parameters of 50 patients with thymoma

Parameter	Number of patients	Percentage (%)
Gender		
Male	21	42
Female	29	58
Symptoms	29	58
Retrosternal pressure	6	12
Chest pain	5	10
Dysphagia	5	10
Dyspnea	3	6
Cough	2	4
Hoarseness	1	2
Involuntary weight loss	4	8
Superior vena cava syndrome	2	4
Myasthenia gravis	14	28
Diagnostics		
CT scan	50	100
PET-CT	25	50
Tumor biopsy	35	70
Neoadjuvant therapy		
Chemotherapy	1	2
Chemoradiation	1	2
Surgery	45	90
Sternotomy	24	53
VATS	15	33
Thoracotomy	6	13
R0 resection	42	93
R1 resection	2	5
R2 resection	1	2
Adjuvant therapy		
Chemoradiation	1	2
Radiotherapy	24	48
Palliative therapy		
Chemotherapy	4	8
Radiotherapy	1	2
Chemoradiation	1	2
Histology		
A	6	12
AB	5	10
B1	8	16
B2	12	24
B3	12	24
C	7	14
Masaoka stage		
I	20	40
IIA	15	30
IIB	3	6
III	6	12
IVA	1	2
IVB	5	10

There was no 30-day mortality. However, seven patients (14 %) developed complications. Intraoperative bleeding occurred in three patients, postoperative ipsilateral pleural empyema in one, progressive mediastinal and soft tissue emphysema in one, and pneumonia in two.

Five (11.1 %) patients out of 45 who had undergone thymectomy developed tumor recurrence. The mean DFS was 58.4 months (ranged 28–77 months). The 5-year DFS was 91.5 %. Up to the end of this study, four of them are still alive; one patient died 92 months after thymectomy due to heart failure. A detailed survey of these 50 patients containing their different treatment modalities according to Masaoka stage is given in Table 2.

**Table 2:** Survey of 50 patients containing their different treatment modalities according to Masaoka stage

Masaoka	Total number	Biopsy	Neoadjuvant treatment	Sternotomy	VATS	Thoracotomy	Adjuvant radiation	Recurrence	Death
I	20	13		11	7	4	2		
IIA	15	9		10	6	1	14	3	2
IIB	3	1		2	2	1	3		
III	6	6	1	5			1	2	1
IVA	1	1	2	1			5		
IVB	5	5					1		2

*VATS* video-assisted thoracic surgery

Palliative therapy was administered in six patients. Definitive radiotherapy due to local inoperability was applied in one case, definitive chemoradiotherapy was done in one patient, and chemotherapy as solo treatment was performed in four patients.

The 5-year OS was 82.3 % and median survival time was 92.1 months (95 % CI 81.8 to 102.3). The 5-year survival rate after thymectomy was 87.2 % and the corresponding median survival was 92.1 months (95 % CI 81.8 to 102.3). No patient was lost to follow-up.

Tumor histology, tumor volume, Masaoka stages I to III, and MG had no significant influence on patient’s survival. At the end of follow-up, 5 out of 50 patients (10 %) had died, whereas only 2 deaths (Masaoka III and IV) were cancer-related. These two patients died after 13 and 12 months, respectively.

## Discussion

The correct treatment of thymoma is different in relation to the pre-therapeutic tumor staging. Surgical resection is of utmost importance in the therapy of non-metastatic thymoma [1]. Chemotherapy and radiotherapy represent further supportive treatment modalities which are applied depending on the individual tumor stage [1, 2].

Preoperative patient selection is indispensable for the appropriate surgical approach. Median sternotomy was the dominant technique in this present analysis which is confirmed by most authors [5]. Although the open technique was used in the majority of patients, we share the argument that VATS-thymectomy is a safe approach achieving comparable results in case of small thymomas without perithymic invasion [8]. Therefore, excellent 5- and 10-year survival rates are reported for resected early-stage thymomas [3, 4]. Complete thymectomy, early-stage disease, and the WHO classification are factors responsible for long-term DFS and better OS [9, 10]. However, these data are in pleasant accordance with our results.

In case of locally advanced disease corresponding to Masaoka III, immediate thymectomy is often precluded and the multimodal approach is demanded [2, 11, 12]. In our opinion, the adequate decision should not be made by the thoracic surgeon alone. There is more responsibility required emphasizing the need for an interdisciplinary tumor board which should address all different aspects related to each individual case as it was already done in our cohort of patients.

In the present study, 25 patients (55.6 %) received adjuvant treatment after surgery [13]. Although there is evidence that a complete resection obviates the need for adjuvant radiotherapy in early-stage disease [3], in our collective, all thymoma stage II underwent postoperative radiation to prevent tumor recurrence as good as possible [14]. In the case of Masaoka stage III, adjuvant therapy was administered due to clear evidence supporting the impact of postoperative radiation [1, 2].

Postoperative tumor recurrence still remains a serious problem. Tumor histology, Masaoka stage, completeness of resection, and the tumor size have been identified as independent predictors of recurrence [15], showing recurrence rates for Masaoka stages II and III between 16 and 26 %, respectively. In our collective, 11 % of the patients had tumor recurrence after a mean DFS of 58.4 months which is in line with recent literature [3, 4]. Among these five patients, tumor recurrence was to be found intrathoracic, confined to the pleura in all cases, although postoperative radiotherapy had been administered. There were neither lymph node metastases nor lung metastases. Surgical management of the recurrence is the treatment of choice [2] and is associated with better outcome compared to non-surgical therapy [16, 17]. In our current analysis, all patients with tumor recurrence were amenable to complete re-resection. However, up to the end of follow-up, one patient died not related to tumor, whereas the remaining four patients are still alive showing no evidence of progression of disease.

It is a matter of fact that associated MG represents a crucial cofactor in approximately 25–33 % of the patients with thymoma influencing considerably the perioperative course [4, 18]. Therefore, every patient in our collective had to undergo preoperative neurologic investigation. In particular, those patients with preoperative MG (*N* = 14; 28 %) were identified and specific medication was administered according to the suggestion given by the neurologist. After thymectomy, MG subsided continuously except in one case. Our remission rate (92.9 %) is in line with the results reported after extended trans-sternal thymectomy [18] underlining the imperative necessity for both meticulous resection and the close teamwork between thoracic surgeon and neurologist in order to optimize the neurologic outcome. In contrast, the incidence of postoperative MG appearing for the first time is considerably less showing up with 1–3 % [19]. The detailed mechanism of this rare and delicate phenomenon remains unclear and is still a matter of debate [20]. In our collective, post-thymectomy MG was diagnosed in only three patients, showing no evidence of preoperative MG. Complete remission could be obtained in two of them by administration of anticholinesterase drugs combined with corticosteroids, plasmapheresis, and immunoglobulin therapy. In the third case, additional redo-surgery with extended resection of all remaining mediastinal fat was done. In this context, extended open thymectomy for patients even without preoperative MG is still recommended [18, 19].

For the sake of completeness, the significance of thymic carcinoma should be briefly discussed at this point. It is beyond all question that complete thymectomy is the mainstay of treatment for non-metastatic thymic carcinoma to maintain disease control and for the prolongation of survival [21, 22], particularly in the early Masaoka stages [23]. In contrast, the value of supportive therapy remains controversial. Whereas some authors have second thoughts concerning the benefit of chemotherapy and radiotherapy [21], other experts conclude that long-term survival can be ensured using a multimodality treatment [22]. Although the number of thymic carcinomas in this study is too small to draw serious conclusions (*N* = 7), their stage-based multimodal treatment was done according to these recent recommendations given by the local tumor board. At the end of follow-up, two of these seven patients had died, whereas only one death was related to tumor progression.

Finally, there are two limitations regarding our current study we want to address. The relatively short time period may represent the first limit, whereas the retrospective nature of the study is the second drawback. This situation is hardly avoidable due to the rarity of the investigated disease (resulting in a small number of patients), in particular in a comparatively small country.

## Conclusions

Nevertheless, we are able to corroborate that thymectomy still remains the mainstay in the treatment of non-metastatic thymoma and should therefore be performed whenever feasible. Complete resection is definitely essential for disease control and long-term survival of patients. Thus, meticulous surgery with complete removal of the thymus gland and the associated fat is required in order to prevent tumor recurrence and postoperative MG, respectively. We want to emphasize that close multidisciplinary teamwork between thoracic surgery, oncology, radiotherapy, and neurology is mandatory in order to optimize the neurologic outcome and to prolong postoperative survival.
